# Identification of gene expression patterns using planned linear contrasts

**DOI:** 10.1186/1471-2105-7-245

**Published:** 2006-05-05

**Authors:** Hao Li, Constance L Wood, Yushu Liu, Thomas V Getchell, Marilyn L Getchell, Arnold J Stromberg

**Affiliations:** 1 Department of Statistics, University of Kentucky, 817 Patterson Office Tower, Lexington, KY40536-0027, USA; 2 Department of Physiology, College of Medicine, Lexington, KY40536-0298, USA; 3 Department of Anatomy and Neurobiology, College of Medicine, Lexington, KY40536-0298, USA; 4 309 Sanders-Brown Center on Aging, University of Kentucky Medical Center, Lexington, KY40536-0230, USA

## Abstract

**Background:**

In gene networks, the timing of significant changes in the expression level of each gene may be the most critical information in time course expression profiles. With the same timing of the initial change, genes which share similar patterns of expression for any number of sampling intervals from the beginning should be considered co-expressed at certain level(s) in the gene networks. In addition, multiple testing problems are complicated in experiments with multi-level treatments when thousands of genes are involved.

**Results:**

To address these issues, we first performed an ANOVA F test to identify significantly regulated genes. The Benjamini and Hochberg (BH) procedure of controlling false discovery rate (FDR) at 5% was applied to the P values of the F test. We then categorized the genes with a significant F test into 4 classes based on the timing of their initial responses by sequentially testing a complete set of orthogonal contrasts, the reverse Helmert series. For genes within each class, specific sequences of contrasts were performed to characterize their general 'fluctuation' shapes of expression along the subsequent sampling time points. To be consistent with the BH procedure, each contrast was examined using a stepwise Studentized Maximum Modulus test to control the gene based maximum family-wise error rate (MFWER) at the level of *α*_*new *_determined by the BH procedure. We demonstrated our method on the analysis of microarray data from murine olfactory sensory epithelia at five different time points after target ablation.

**Conclusion:**

In this manuscript, we used planned linear contrasts to analyze time-course microarray experiments. This analysis allowed us to characterize gene expression patterns based on the temporal order in the data, the timing of a gene's initial response, and the general shapes of gene expression patterns along the subsequent sampling time points. Our method is particularly suitable for analysis of microarray experiments in which it is often difficult to take sufficiently frequent measurements and/or the sampling intervals are non-uniform.

## Background

Recent advances in DNA microarray technologies have made it possible to investigate the transcriptional portion of gene networks in a variety of organisms. When microarray experiments are performed to monitor gene expression over time, researchers can address questions concerning the detection of the cellular processes underlying the observed regulatory effects, inference of regulatory networks and, ultimately, assignment of functions to the genes analyzed in the time courses.

There is a natural connection between gene function and gene expression. Based on our understanding of cellular processes, genes that are contained in a particular pathway, or respond to a common internal or external stimulus, should be co-regulated and consequently, should show similar patterns of expression. Therefore, identifying patterns of gene expression and grouping genes into expression classes may provide much greater insight into their biological functions. A large group of statistical methods, generally referred to as "cluster analysis", have been developed to identify genes that behave similarly across a range of experimental conditions, including time courses. These statistical algorithms can be divided into two classes, depending on whether they are based on 'similarity' measures or not. Methods based on 'similarity' measures rely on defining a distance (or 'dissimilarity') between gene expression vectors; Euclidean distance and/or the Pearson correlation coefficient are the two most commonly used distance measures. Examples of similarity measures-based methods are hierarchical clustering [1], k-means [2], self-organization maps (SOM) [3,4], and support vector machine (SVM) [5]. These methods do not consider the temporal structure of the data when used to analyze time-course experiments. In addition, some methods could confuse the clusters because the actual expression patterns of the genes themselves become less relevant as clusters grow in size [6].

The clustering methods in the second class are based on statistical models, without defining a 'similarity' measure. Using statistical models to represent clusters changes the question from how close two data points are to how likely a given data point is under the model. Such clustering methods are more commonly used to analyze time-course microarray experiments. Examples of such methods are based on cubic spline [7], ANOVA model [8], autoregressive curves [9], first-order kinetics [10], Hidden Markov Models [11,12], Bayesian model average [13], order-restricted inference methodology [14], and Gaussian Mixture Models [15-19]. Such approaches may be restricted either by the rigorous assumptions of the stochastic models [9,11,12], or by the small number of time points and non-uniform sampling intervals in gene expression data [7,9,10].

In gene networks, the level of expression of individual genes changes based on their functional position in the network. Therefore, the most critical information in time course expression profiles is the timing of the changes in expression level for each gene [10], and secondarily is the general shape of its expression pattern [20,21]. In addition, different genes will be activated or inactivated at each level of a gene network. Therefore it may not be reasonable to expect that the expression levels of those co-expressed genes will go up and down concordantly all the way through the entire sampling period. With the same timing of initial change, genes which share similar pattern of expression for any number of sampling intervals from the beginning should be considered co-expressed at certain level(s) in the gene network. However, statistical methods to analyze these patterns have not yet been reported.

Attention to the multiplicity problem in gene expression analysis has been increasing. Numerous methods are available for controlling the family-wise type I error rate (FWER). Since microarray experiments are frequently exploratory in nature and the sample sizes are usually small, Benjamini and Hochberg [22] suggested a potentially more powerful procedure, the false discovery rate (FDR), to control the expected proportion of errors among the identified differentially expressed genes. A number of studies for controlling FDR have followed [23-29]. In microarray experiments with multi-level treatments, the multiple testing problems are two dimensional. Not only are thousands of genes involved, but for each gene, either pre-selected contrasts or post-hoc comparisons may be needed to characterize its expression pattern. There are very few studies that have investigated how to deal with such multiple-testing problems in the microarray literature [30].

In this manuscript, we propose a different strategy based on planned linear contrasts (pre-selected contrasts) for the analysis of time-course microarray experiments. Specifically, our approach takes into consideration the temporal order in the data, including the timing of a gene's initial response and the general shapes of gene expression patterns along the subsequent sampling time points. Our methods are particularly suitable for analysis of microarray experiments in which it is often difficult to take sufficiently frequent measurements and/or the sampling intervals are non-uniform. We demonstrated our method on the analysis of microarray data from murine olfactory sensory epithelia at five different time points after target ablation.

## Results

**Figure 1:**
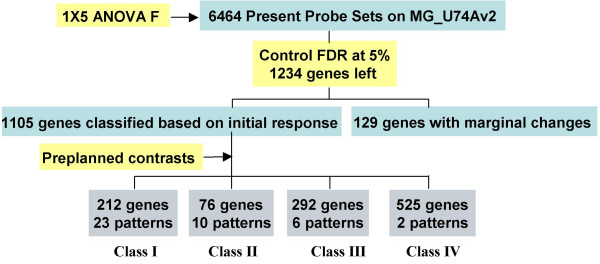
**Flow chart illustrating the statistical procedure to classify gene expression patterns**. A 1 × 5 ANOVA F test was performed for each of the 6464 genes after data filtering. By controlling FDR at 5%, 1234 genes were selected, 1105 of which were clustered into 4 classes based on the timing of their initial significant change in expression level. The fluctuation patterns of genes in each class were examined using planned linear contrasts.

Olfactory sensory neurons (OSNs) detect odors in the ambient environment and transmit the sensory information directly to the brain. The death of OSNs can be induced experimentally by microsurgical removal of their axonal targets in the brain (olfactory bulbectomy, OBX). The temporal regulation of genes associated with the death of OSNs and other cellular processes as a result of OBX can be systematically investigated at 2 hr, 8 hr, 16 hr and 48 hr post-OBX. Based on the statistical methods described (see Methods), 1234 genes were considered to be significant by the procedure of controlling FDR at 5% for multiple testing across genes. The largest P-value considered to be significant was 0.009545 as determined by the FDR procedure. The temporal regulation of these 1234 genes fell into four distinct classes based on the first significant change in their temporal profile that occurred at either 2 hr (Class I), 8 hr (Class II), 16 hr (Class III), or 48 hr (Class IV) post-OBX. Among the 1234 genes (Figure 1), 212 were grouped into Class I in which the differential expression of these genes was detected as early as 2 hours after target ablation. Seventy-six genes were grouped into Class II, 292 genes whose expression level first changed at 16 hr post-OBX into Class III, and 525 genes whose expression level first changed at 48 hours after the surgery were grouped into Class IV. The remaining 129 genes did not pass our selection criteria although their ANOVA F tests were significant.

**Figure 2:**
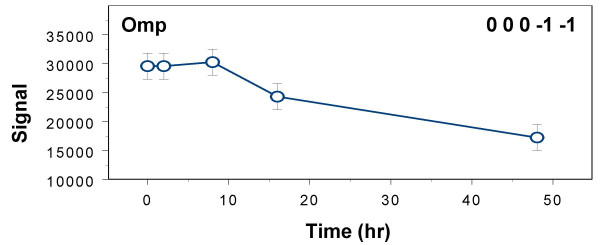
**A simple diagram to illustrate the expressionpattern of the gene *Omp***. Mean hybridization signals (± SD) at each time point were plotted. The expression of *Omp *was unchanged at 2 hr and 8 hr following OBX. Its first significant change in expression was a down-regulation at 16 hr post-OBX. Its expression continued to decrease at 48 hr. The fluctuation pattern of *Omp *expression was indexed by (0 0 0 -1 -1), shown in the upper right corner of the graph. Diagrams in the following figures were plotted similarly.

The expression level of the gene for olfactory marker protein *Omp*, which is expressed in mature OSNs, was unchanged at 2 hr and 8 hr following OBX. The initial change, a down-regulation at 16 hr post-OBX, indicated that degeneration was evident between 8 hr and 16 hr post-OBX (Figure 2). The significant down-regulation of *Omp *(p = 1.6E-5, Table 1) continued to the 48 hr time-point that was accompanied by a -1.6 FC in OMP mRNA, indicating degenerative changes in OSNs accompanying their cell death.

**Figure 3:**
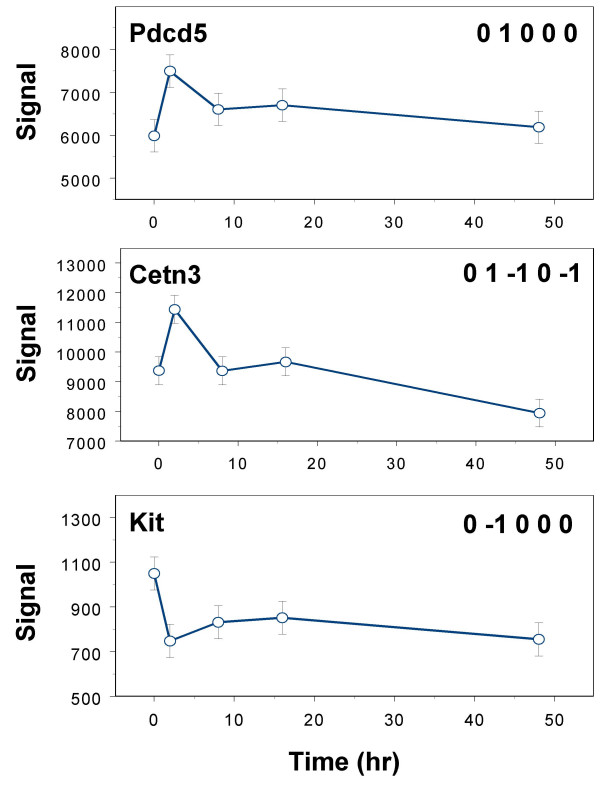
**Example diagrams to illustrate expression patterns of genes in Class I**. Mean signals (± SD) for genes *Pdcd5*, *Cetn3*, and *Kit *from Class I were plotted. Their expression levels were significantly altered at as early as 2 hr after OBX. Their subsequent expression patterns were different, which was indicated by the indices in each panel.

**Figure 4:**
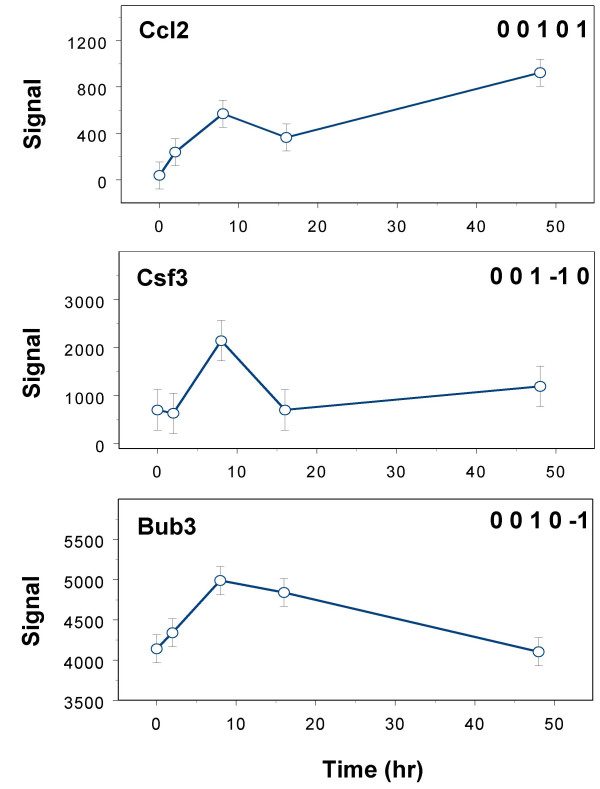
**Example diagrams to illustrate expression patterns of genes in Class II**. Expression patterns of genes *Ccl2*, *Csf3*, and *Bub3 *from Class II were significantly altered initially at 8 hr after OBX, and each of these genes has a different fluctuation index.

**Figure 5:**
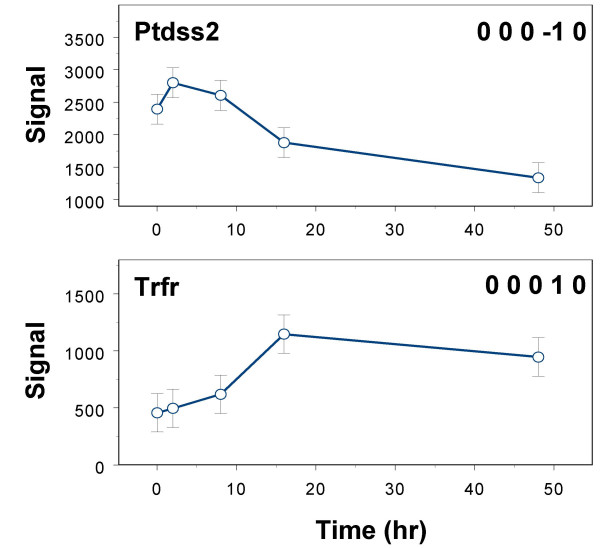
**Example diagrams to illustrate expression pattern of genes in Class III**. Expression patterns of genes *Ptdss2*, and *Tfrc *from Class III, which are unchanged at 2 hr and 8 hr, were first significantly altered at 16 hr after OBX, and each had a different fluctuation index.

**Figure 6:**
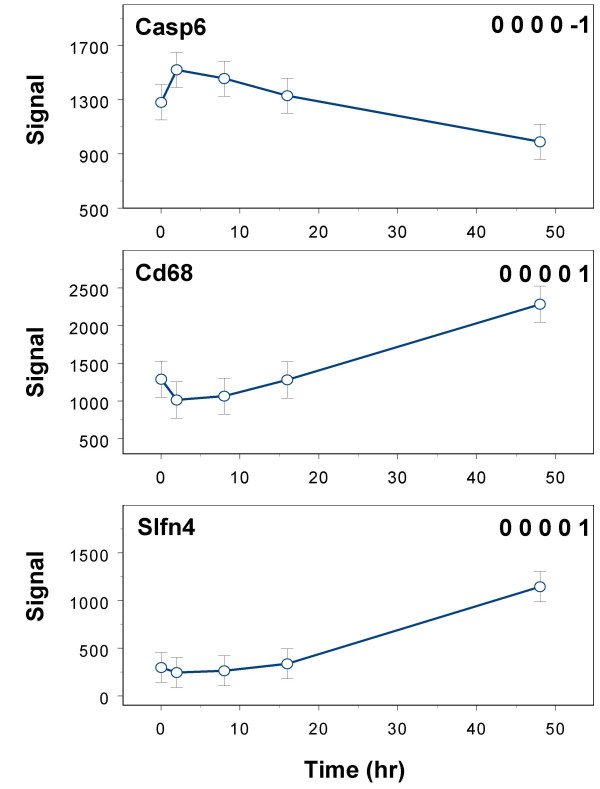
**Example diagrams to illustrate expression patterns of genes in Class IV**. Expression patterns of genes *Casp6*, *Cd68 *and *Slfn4 *from Class IV, were unchanged until the last time point sampled, 48 hr after OBX.

The genes for programmed cell death 5 (*Pdcd5*), centrin 3 (*Cetn3*), and *Kit *are examples of Class I genes that showed their first significant change in temporal expression at 2 hr post-OBX, with *Pdcd5 *and *Cetn3 *being up-regulated and *Kit *being down-regulated (Figure 3). In contrast, Class II genes showed their first significant change in temporal expression at 16 hr post-OBX (Figure 4); they included the genes for chemokine (C-C motif) ligand 2 (*Ccl2*), colony stimulating factor 3 (*Csf3*), and budding uninhibited by benzimidazoles 3 homolog (*Bub3*) that were up-regulated simultaneously. The genes for phosphatidylserine synthase 2 (*Ptdss2*) and the transferrin receptor (*Tfrc*) are examples of Class III genes that showed their first significant change in temporal expression at 16 hr post-OBX, with *Ptdss2 *and *Tfrc *down-regulated and up-regulated respectively (Figure 5). The genes identified statistically as Class IV genes were initially quiescent until their first significant change in expression at 48 hr post-OBX (Figure 6) as shown by the genes for caspase 6 (*Casp6*), CD68 antigen (*Cd68*), and schlafen 4 (*Slfn4*). From a functional perspective, the regulation of the genes for *Pdcd5*, *Ptdss2*, and *Casp6 *at 2 hr, 16 hr, and 48 hr respectively suggested that the molecular mechanisms associated with OSN degeneration and cell death occurred over a 2d time frame that is consistent with the systematic down-regulation of the gene for *Omp*. The up-regulation of the genes for *Ccl2 *and *Cd68 *at 8 hr and 48 hr respectively suggested the expression of macrophage chemoattractant protein-1 (CCL2) by resident and recruited macrophages identified phenotypically with CD68 antibody that indicate the delivery of bioactive molecules associated with the earliest regeneration of the sensory epithelium. The genes for *Kit*, *Csf3*, *Bub3*, and *Slfn4 *are broadly defined as having growth factor activity, which suggested that molecular mechanisms associated with the transformation of progenitor cells into mature OSNs through the proliferative stages of the cell cycle was initiated within 2 hr of OBX and continued throughout the following 48 hr. The results of our statistical and bioinformatics analyses clearly indicate that the categorization of genes into four Classes based on their first significant temporal regulatory event has biological relevance at the cellular level in this neurosensory tissue.

**Figure 7:**
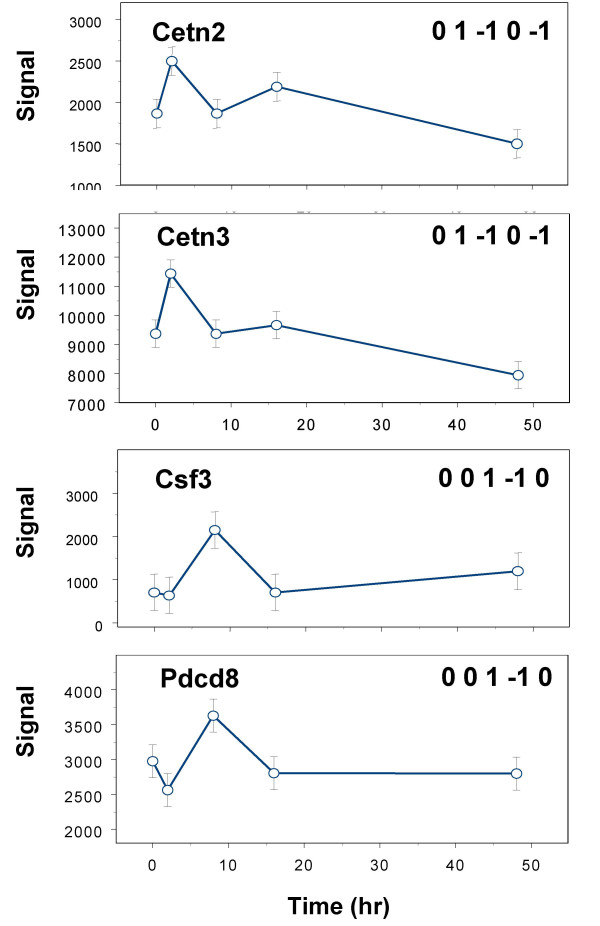
**Genes shared the same expression pattern were grouped together**. Expression patterns of genes *Cetn2 and Cetn3 *from Class I are the same and therefore they were grouped together. Another example is genes Csf3 and Pdcd8 from Class II which were put into one group because of the same expression pattern.

Genes in each class share the same timing of their earliest significant change in expression. The expression pattern of each gene at subsequent time points may vary. We therefore can further cluster genes in each class into subgroups based on their subsequent expression patterns or 'fluctuation patterns'. For genes in Class I (Figure 1), there theoretically may as many as 54 fluctuation patterns. In our example study, we found 23 different patterns in this class. There were 10, 6, and 2 patterns for genes in Class II, III, and IV respectively. We can use simple diagrams to illustrate these patterns and a series of characters (1, 0, -1) to index their expression patterns as described in the Methods. For example, the fluctuation pattern of *Omp *expression (Figure 2) can be represented by (0 0 0 -1 -1), and the expression of *Pdcd5 *can be indicated by (0 1 0 0 0) (Figure 3). Genes with the same fluctuation patterns will be finally grouped into the same group (Figure 7). For example, gene Cetn2 and Cetn3 shared the same expression pattern and can be grouped together. This pattern was indicated by (0 1 -1 0 -1). Genes Csf3 and Pdcd8 had their initial responses at hr16 and shared the same fluctuation pattern. These two genes can be classified into another group indicated as (0 0 1 -1 0). Genes CD68 and Slfn4 (Figure 6) can also form a cluster for the same reason.

## Discussion

In this study, we adopted linear models to describe our data and used planned linear contrasts to analyze time-course microarray experiments. We identified 1234 genes with significant changes in expression in a microarray study of murine olfactory epithelium, and 1105 of them were grouped into 4 classes based on the timing of their initial changes. We further categorized these 1105 genes into 41 fluctuation patterns. We also used simple diagrams to illustrate these fluctuation patterns and a series of characters (1, 0, -1) to index these patterns. Although the ANOVA F tests were significant, 129 genes cannot be grouped into any of these 4 classes based on our criteria. A significant ANOVA F test among a group of means indicates that the largest contrast among all possible contrasts is significant. Therefore, a gene with a significant F test does not necessarily have a significant selected contrast. Therefore the expression patterns of these genes should be interpreted carefully.

The critical value |Mαnew,m−2,v|
 MathType@MTEF@5@5@+=feaafiart1ev1aaatCvAUfKttLearuWrP9MDH5MBPbIqV92AaeXatLxBI9gBaebbnrfifHhDYfgasaacH8akY=wiFfYdH8Gipec8Eeeu0xXdbba9frFj0=OqFfea0dXdd9vqai=hGuQ8kuc9pgc9s8qqaq=dirpe0xb9q8qiLsFr0=vr0=vr0dc8meaabaqaciaacaGaaeqabaqabeGadaaakeaadaabdaqaaiabd2eannaaBaaaleaaiiGacqWFXoqydaWgaaadbaGaemOBa4MaemyzauMaem4DaChabeaaliabcYcaSiabd2gaTjabgkHiTiabikdaYiabcYcaSiabdAha2bqabaaakiaawEa7caGLiWoaaaa@3DAB@ used to select significant contrasts is the uniform upper bound for testing a complete set of contrasts regardless of the correlation structure among these contrasts. It is a conservative approach. For planned linear contrasts, the most powerful bound can be found based on the correlation structure of these contrasts [31,32]. In general, the most powerful bound can't be obtained without knowing the correlation structure among the contrasts [33]. The uniform bound, however, can be obtained from testing a complete set of orthogonal contrasts using the Studentized Maximum Modulus Distribution [34]. In practice, although a little bit conservative, it is straightforward to use this uniform bound to test all contrasts especially when the number of different combinations of contrasts is large.

Our methods emphasized the relative differences between adjacent sampling time points and the direction of the differences. The information about exact magnitudes of gene expressed at each time point was not included in our methods. For example, two genes may have the same pattern index 0 1 -1 0 0, but the magnitude of changes for the two genes may be dramatically different. Therefore, even for genes in the same index groups, their expression patterns should be examined with care.

The temporal order in the data was considered in our methods by the selection sequence but was not parameterized in our model. The information about the differences among sampling intervals were also ignored in our analysis. With small sample sizes and non-uniform sampling intervals, which are very common in biomedical research, our methods may be more straightforward and robust than those commonly in use. With large sample sizes and relative uniform sampling intervals, other methods, such as regression analysis, mixture models, or autoregressive models can be applied.

## Conclusion

Linear models were adopted to describe microarray data, and sequences of planned linear contrasts were used to group genes into different expression patterns based on their initial and subsequent changes in expression. Our methods are particularly suitable for analysis of microarray experiments in which it is often difficult to take sufficiently frequent measurements and/or the sampling intervals are non-uniform. Our methods can also be extended to designs with more than one factor.

## Methods

### Microarray experiments

The goal of this study was to investigate the induction of gene regulation at short time intervals (2, 8, 16, and 48 hrs) following deafferentation of olfactory sensory neurons by target ablation (olfactory bulbectomy, OBX) compared with sham controls [35]. Total RNA was isolated from the olfactory epithelium of 3 male mice per time point (1 GeneChip/mouse). Following hybridization with Affymetrix GeneChips MG U74Av2, 3 chips per time point (a total of 15 GeneChips), the signal intensities were generated by Affymetrix Microarray Suite v5.0.

In our study, all positive control genes and genes that resulted in "absent" calls for all chips across all time points were removed from further analysis. If there was no evidence that these genes were expressed in any of the samples, then these genes can be removed to reduce problems associated with multiple comparisons. Other methods of removing low intensity points were also suggested by Bolstad *et al*., 2003[36]. All ESTs were also removed from the analysis because the primary aim of these experiments was to identify known genes that were differentially regulated; eliminating ESTs further reduced problems with multiple comparisons. After data filtering steps, 6464 genes remained, and the background-corrected intensities of these genes were subjected to further statistical analyses.

### Algorithm and analysis

#### Statistical model

We use a linear model to describe the experiment. Let **Y**_**g **_be the vector of observed expression levels for gene g, g = 1, ..., 6464 then

Y_g _= Xβ_g _+ *ε*_g_

where **X **is the matrix of known constants, **β**_**g **_= (*μ*_*g*1_, *μ*_*g*2_, ..., *μ*_*gm*_), and *m *is the number of time points (*m *= 5 in this study). ***ε***_***g ***_is the random error, and we assume ***ε***_***g ***_~ **MVN(0, *σ*_*g*_^2^*I*)**.

#### Reverse Helmert series

A contrast is a linear combination of parameters for which the coefficients sum to zero. A complete set of orthogonal contrasts is a set of k-1 contrasts in k treatments (or treatment combinations) which provides a complete partitioning of the variability among parameters into mutually exclusive and exhaustive parts. Each contrast in such a set is orthogonal to every other remaining one [37]. One commonly used complete set of orthogonal contrasts is the reverse Helmert series, in which one treatment group is compared with the average of all remaining treatment groups. Subsequent contrasts eliminate the first group and then proceed by comparing one of the remaining groups to the average of the other remaining groups, as show below:

Lβ=(1−10.01212−1.0....01m−11m−11m−1.−1)(μ1μ2..μm)
 MathType@MTEF@5@5@+=feaafiart1ev1aaatCvAUfKttLearuWrP9MDH5MBPbIqV92AaeXatLxBI9gBaebbnrfifHhDYfgasaacH8akY=wiFfYdH8Gipec8Eeeu0xXdbba9frFj0=OqFfea0dXdd9vqai=hGuQ8kuc9pgc9s8qqaq=dirpe0xb9q8qiLsFr0=vr0=vr0dc8meaabaqaciaacaGaaeqabaqabeGadaaakeaaieWacqWFmbatiiWacqGFYoGycqGH9aqpdaqadaqaauaabeqaeuaaaaaabaGaeGymaedabaGaeyOeI0IaeGymaedabaGaeGimaadabaGaeiOla4cabaGaeGimaadabaWaaSaaaeaacqaIXaqmaeaacqaIYaGmaaaabaWaaSaaaeaacqaIXaqmaeaacqaIYaGmaaaabaGaeyOeI0IaeGymaedabaGaeiOla4cabaGaeGimaadabaGaeiOla4cabaGaeiOla4cabaGaeiOla4cabaGaeiOla4cabaGaeGimaadabaWaaSaaaeaacqaIXaqmaeaacqWGTbqBcqGHsislcqaIXaqmaaaabaWaaSaaaeaacqaIXaqmaeaacqWGTbqBcqGHsislcqaIXaqmaaaabaWaaSaaaeaacqaIXaqmaeaacqWGTbqBcqGHsislcqaIXaqmaaaabaGaeiOla4cabaGaeyOeI0IaeGymaedaaaGaayjkaiaawMcaamaabmaabaqbaeqabuqaaaaabaacciGae0hVd02aaSbaaSqaaiabigdaXaqabaaakeaacqqF8oqBdaWgaaWcbaGaeGOmaidabeaaaOqaaiabc6caUaqaaiabc6caUaqaaiab9X7aTnaaBaaaleaacqWGTbqBaeqaaaaaaOGaayjkaiaawMcaaaaa@5FB4@

One of the advantages of the Reverse Helmert contrasts is that these contrasts are orthogonal and, hence, contrasts among the sample means are uncorrelated. Basing tests on uncorrelated contrasts avoids the problems inherent in interpreting conditional tests. Adjacent Differences(AD) are sometimes used to identify the point at which initial gene expression occurs. However, these contrasts are not orthogonal and consecutive contrasts have a correlation of 0.5. Consequently, the probability of identifying the correct threshold is lower for AD than for the Reverse Helmert Contrasts.

#### Clustering genes based on the timing of their initial responses

The reverse Helmert series can test the following *m*-1 hypotheses sequentially:

H10:μ1−μ2=0H20:μ1+μ22−μ3=0........Hs0:μ1+μ2+...+μm−1m−1−μm=0
 MathType@MTEF@5@5@+=feaafiart1ev1aaatCvAUfKttLearuWrP9MDH5MBPbIqV92AaeXatLxBI9gBaebbnrfifHhDYfgasaacH8akY=wiFfYdH8Gipec8Eeeu0xXdbba9frFj0=OqFfea0dXdd9vqai=hGuQ8kuc9pgc9s8qqaq=dirpe0xb9q8qiLsFr0=vr0=vr0dc8meaabaqaciaacaGaaeqabaqabeGadaaakqaabeqaaiabdIeainaaBaaaleaacqaIXaqmcqaIWaamaeqaaOGaeiOoaOdcciGae8hVd02aaSbaaSqaaiabigdaXaqabaGccqGHsislcqWF8oqBdaWgaaWcbaGaeGOmaidabeaakiabg2da9iabicdaWaqaaiabdIeainaaBaaaleaacqaIYaGmcqaIWaamaeqaaOGaeiOoaOZaaSaaaeaacqWF8oqBdaWgaaWcbaGaeGymaedabeaakiabgUcaRiab=X7aTnaaBaaaleaacqaIYaGmaeqaaaGcbaGaeGOmaidaaiabgkHiTiab=X7aTnaaBaaaleaacqaIZaWmaeqaaOGaeyypa0JaeGimaadabaGaeiOla4IaeiOla4IaeiOla4IaeiOla4IaeiOla4IaeiOla4IaeiOla4IaeiOla4cabaGaemisaG0aaSbaaSqaaiabdohaZjabicdaWaqabaGccqGG6aGodaWcaaqaaiab=X7aTnaaBaaaleaacqaIXaqmaeqaaOGaey4kaSIae8hVd02aaSbaaSqaaiabikdaYaqabaGccqGHRaWkcqGGUaGlcqGGUaGlcqGGUaGlcqGHRaWkcqWF8oqBdaWgaaWcbaGaemyBa0MaeyOeI0IaeGymaedabeaaaOqaaiabd2gaTjabgkHiTiabigdaXaaacqGHsislcqWF8oqBdaWgaaWcbaGaemyBa0gabeaakiabg2da9iabicdaWaaaaa@7062@

Genes will be partitioned into *m*-1 classes based on the testing results of *H*_10 _~ *H*_*s*0_, where *s *= *m*-1. Class 1 contains genes that reject *H*_10_; genes that reject *H*_20 _from the remaining list are grouped into class 2, and so on; Class *s *includes genes that reject *H*_*s*0 _without rejecting the previous *s*-1 hypotheses. Therefore Genes in Class 1 are considered to be early responding genes whose expression levels are significantly altered during the first sampling interval, that is, at the 2^nd ^sampling time point. Genes that do not change their expression levels until the 3^rd ^sampling time point are collected in Class 2, and so on. As indicated by the described partition process, genes within a class share the same timing of onset or cessation of expression.

#### Clustering genes within a class

Genes in each of these above *m*-1 classes can be further classified based on their 'fluctuation' shapes at the subsequent sampling points. For gene g in class *j*, where *j *= 1, 2, ..., *s*, the following *s*-*j *contrasts are needed,

Hgj,1:μgj+1−μgj+2=0
 MathType@MTEF@5@5@+=feaafiart1ev1aaatCvAUfKttLearuWrP9MDH5MBPbIqV92AaeXatLxBI9gBaebbnrfifHhDYfgasaacH8akY=wiFfYdH8Gipec8Eeeu0xXdbba9frFj0=OqFfea0dXdd9vqai=hGuQ8kuc9pgc9s8qqaq=dirpe0xb9q8qiLsFr0=vr0=vr0dc8meaabaqaciaacaGaaeqabaqabeGadaaakeaacqWGibasdaqhaaWcbaGaem4zaCgabaGaemOAaOMaeiilaWIaeGymaedaaOGaeiOoaOdcciGae8hVd02aa0baaSqaaiabdEgaNbqaaiabdQgaQjabgUcaRiabigdaXaaakiabgkHiTiab=X7aTnaaDaaaleaacqWGNbWzaeaacqWGQbGAcqGHRaWkcqaIYaGmaaGccqGH9aqpcqaIWaamaaa@4347@

Hgj,k:(μgj+k−μgj+k+1=0if reject Hgj,k−1μgj+k−1+μgj+k2−μgJ=k+1=0if fail to reject Hgj,k−1)k=2,…s−j
 MathType@MTEF@5@5@+=feaafiart1ev1aaatCvAUfKttLearuWrP9MDH5MBPbIqV92AaeXatLxBI9gBaebbnrfifHhDYfgasaacH8akY=wiFfYdH8Gipec8Eeeu0xXdbba9frFj0=OqFfea0dXdd9vqai=hGuQ8kuc9pgc9s8qqaq=dirpe0xb9q8qiLsFr0=vr0=vr0dc8meaabaqaciaacaGaaeqabaqabeGadaaakeaafaqabeqacaaabaGaemisaG0aa0baaSqaaiabdEgaNbqaaiabdQgaQjabcYcaSiabdUgaRbaakiabcQda6maabmaabaqbaeqabiGaaaqaaGGaciab=X7aTnaaDaaaleaacqWGNbWzaeaacqWGQbGAcqGHRaWkcqWGRbWAaaGccqGHsislcqWF8oqBdaqhaaWcbaGaem4zaCgabaGaemOAaOMaey4kaSIaem4AaSMaey4kaSIaeGymaedaaOGaeyypa0JaeGimaadabaGaeeyAaKMaeeOzayMaeeiiaaIaeeOCaiNaeeyzauMaeeOAaOMaeeyzauMaee4yamMaeeiDaqNaeeiiaaIaemisaG0aa0baaSqaaiabdEgaNbqaaiabdQgaQjabcYcaSiabdUgaRjabgkHiTiabigdaXaaaaOqaamaalaaabaGae8hVd02aa0baaSqaaiabdEgaNbqaaiabdQgaQjabgUcaRiabdUgaRjabgkHiTiabigdaXaaakiabgUcaRiab=X7aTnaaDaaaleaacqWGNbWzaeaacqWGQbGAcqGHRaWkcqWGRbWAaaaakeaacqaIYaGmaaGaeyOeI0Iae8hVd02aa0baaSqaaiabdEgaNbqaaiabdQeakjabg2da9iabdUgaRjabgUcaRiabigdaXaaakiabg2da9iabicdaWaqaaiabbMgaPjabbAgaMjabbccaGiabbAgaMjabbggaHjabbMgaPjabbYgaSjabbccaGiabbsha0jabb+gaVjabbccaGiabbkhaYjabbwgaLjabbQgaQjabbwgaLjabbogaJjabbsha0jabbccaGiabdIeainaaDaaaleaacqWGNbWzaeaacqWGQbGAcqGGSaalcqWGRbWAcqGHsislcqaIXaqmaaaaaaGccaGLOaGaayzkaaaabaGaem4AaSMaeyypa0dcbiGae4NmaiJaeiilaWIaeSOjGSKaem4CamNaeyOeI0IaemOAaOgaaaaa@A0BE@

Therefore, a specific sequence of *m*-1 hypotheses will be performed for each gene to determine its expression pattern. Let λ^gj,k
 MathType@MTEF@5@5@+=feaafiart1ev1aaatCvAUfKttLearuWrP9MDH5MBPbIqV92AaeXatLxBI9gBaebbnrfifHhDYfgasaacH8akY=wiFfYdH8Gipec8Eeeu0xXdbba9frFj0=OqFfea0dXdd9vqai=hGuQ8kuc9pgc9s8qqaq=dirpe0xb9q8qiLsFr0=vr0=vr0dc8meaabaqaciaacaGaaeqabaqabeGadaaakeaaiiGacuWF7oaBgaqcamaaDaaaleaacqWGNbWzaeaacqWGQbGAcqGGSaalcqWGRbWAaaaaaa@3397@ be the unbiased estimate of the contrast corresponding to the hypothesis Hgj,k
 MathType@MTEF@5@5@+=feaafiart1ev1aaatCvAUfKttLearuWrP9MDH5MBPbIqV92AaeXatLxBI9gBaebbnrfifHhDYfgasaacH8akY=wiFfYdH8Gipec8Eeeu0xXdbba9frFj0=OqFfea0dXdd9vqai=hGuQ8kuc9pgc9s8qqaq=dirpe0xb9q8qiLsFr0=vr0=vr0dc8meaabaqaciaacaGaaeqabaqabeGadaaakeaacqWGibasdaqhaaWcbaGaem4zaCgabaGaemOAaOMaeiilaWIaem4AaSgaaaaa@32E5@, in a balanced experiment with sample size *n *in each treatment group, the statistic

Tgj,k=λ^gj,kMSEg∑i=1mcig2n~tv
 MathType@MTEF@5@5@+=feaafiart1ev1aaatCvAUfKttLearuWrP9MDH5MBPbIqV92AaeXatLxBI9gBaebbnrfifHhDYfgasaacH8akY=wiFfYdH8Gipec8Eeeu0xXdbba9frFj0=OqFfea0dXdd9vqai=hGuQ8kuc9pgc9s8qqaq=dirpe0xb9q8qiLsFr0=vr0=vr0dc8meaabaqaciaacaGaaeqabaqabeGadaaakeaacqWGubavdaqhaaWcbaGaem4zaCgabaGaemOAaOMaeiilaWIaem4AaSgaaOGaeyypa0ZaaSaaaeaaiiGacuWF7oaBgaqcamaaDaaaleaacqWGNbWzaeaacqWGQbGAcqGGSaalcqWGRbWAaaaakeaadaGcaaqaaiabd2eanjabdofatjabdweafnaaBaaaleaacqWGNbWzaeqaaOWaaSaaaeaadaaeWbqaaiabdogaJnaaDaaaleaacqWGPbqAcqWGNbWzaeaacqaIYaGmaaaabaGaemyAaKMaeyypa0JaeGymaedabaGaemyBa0ganiabggHiLdaakeaacqWGUbGBaaaaleqaaaaakiabc6ha+Hqadiab+rha0naaBaaaleaacqGF2bGDaeqaaaaa@524B@

where *c*_*i *_is the ith coefficient for the contrast, and i = 1, ..., *m*. MSE_g _is the usual unbiased estimate of σg2
 MathType@MTEF@5@5@+=feaafiart1ev1aaatCvAUfKttLearuWrP9MDH5MBPbIqV92AaeXatLxBI9gBaebbnrfifHhDYfgasaacH8akY=wiFfYdH8Gipec8Eeeu0xXdbba9frFj0=OqFfea0dXdd9vqai=hGuQ8kuc9pgc9s8qqaq=dirpe0xb9q8qiLsFr0=vr0=vr0dc8meaabaqaciaacaGaaeqabaqabeGadaaakeaaiiGacqWFdpWCdaqhaaWcbaGaem4zaCgabaGaeGOmaidaaaaa@30EC@, and *v *= N-*m *is the error degree of freedom (df), where N = *mn*.

#### Indexing gene expression patterns

Let the state of the first observation be 0, for gene g, its expression profile can be transformed into a sequence of expression fluctuation as follows:

Sgj,k={1if k≥j, reject Hgj,k, and λ^gj,k<00if k<j or fail to reject Hgj,k−1if k≥j, reject Hgj,k, and λ^gj,k>0}j=1,…,s,k=1,…,s
 MathType@MTEF@5@5@+=feaafiart1ev1aaatCvAUfKttLearuWrP9MDH5MBPbIqV92AaeXatLxBI9gBaebbnrfifHhDYfgasaacH8akY=wiFfYdH8Gipec8Eeeu0xXdbba9frFj0=OqFfea0dXdd9vqai=hGuQ8kuc9pgc9s8qqaq=dirpe0xb9q8qiLsFr0=vr0=vr0dc8meaabaqaciaacaGaaeqabaqabeGadaaakeaafaqabeqadaaabaGaee4uam1aa0baaSqaaiabdEgaNbqaaiabdQgaQjabcYcaSiabdUgaRbaakiabg2da9maacmaabaqbaeqabmGaaaqaaiabigdaXaqaaiabbMgaPjabbAgaMjabbccaGiabdUgaRjabgwMiZkabdQgaQjabcYcaSiabbccaGiabbkhaYjabbwgaLjabbQgaQjabbwgaLjabbogaJjabbsha0jabbccaGiabdIeainaaDaaaleaacqWGNbWzaeaacqWGQbGAcqGGSaalcqWGRbWAaaGccqGGSaalcqqGGaaicqqGHbqycqqGUbGBcqqGKbazcqqGGaaiiiGacuWF7oaBgaqcamaaDaaaleaacqWGNbWzaeaacqWGQbGAcqGGSaalcqWGRbWAaaGccqGH8aapcqaIWaamaeaacqaIWaamaeaacqqGPbqAcqqGMbGzcqqGGaaicqWGRbWAcqGH8aapcqWGQbGAcqqGGaaicqqGVbWBcqqGYbGCcqqGGaaicqqGMbGzcqqGHbqycqqGPbqAcqqGSbaBcqqGGaaicqqG0baDcqqGVbWBcqqGGaaicqqGYbGCcqqGLbqzcqqGQbGAcqqGLbqzcqqGJbWycqqG0baDcqqGGaaicqWGibasdaqhaaWcbaGaem4zaCgabaGaemOAaOMaeiilaWIaem4AaSgaaaGcbaGaeyOeI0IaeGymaedabaGaeeyAaKMaeeOzayMaeeiiaaIaem4AaSMaeyyzImRaemOAaOMaeiilaWIaeeiiaaIaeeOCaiNaeeyzauMaeeOAaOMaeeyzauMaee4yamMaeeiDaqNaeeiiaaIaemisaG0aa0baaSqaaiabdEgaNbqaaiabdQgaQjabcYcaSiabdUgaRbaakiabcYcaSiabbccaGiabbggaHjabb6gaUjabbsgaKjabbccaGiqb=T7aSzaajaWaa0baaSqaaiabdEgaNbqaaiabdQgaQjabcYcaSiabdUgaRbaakiabg6da+iabicdaWaaaaiaawUhacaGL9baaaeaacqWGQbGAcqGH9aqpieGacqGFXaqmcqGGSaalcqWIMaYscqGGSaalcqWGZbWCcqGGSaalaeaacqWGRbWAcqGH9aqpcqGFXaqmcqGGSaalcqWIMaYscqGGSaalcqWGZbWCaaaaaa@BED9@

where *k *= 1, 2, ..., *s *is an index, where *k *= *r *if it is the rth contrast for gene g. S is the transformed value of the gene expression profiles. Thus an *m*-time-point expression profile is transformed into an *m*-1-state sequence of expression fluctuation consisting of a character set (1, 0, -1). Each character in the sequence indicates whether the mean expression level of the gene is significantly up-regulated (1), not altered (0), or significantly down-regulated (-1) at the next time point, while the whole sequence represents the fluctuation pattern of the gene expression. Besides the pattern in which the gene's expression level is unchanged throughout the entire sampling period, there are at most 2 × 3^*m*-*k*-1 ^fluctuation patterns for genes in Class *k*. There are no more than 3^*m*-1 ^patterns of expression in total in an *m*-time-point microarray experiment.

#### Multiple testing control

An ANOVA F test was performed for each gene to identify the differentially expressed genes. This F test is testing the hypothesis *μ*_*g*1 _= *μ*_*g*2_, ..., = *μ*_*gm*_, which is equivalent to test the composite hypothesis ***Lβ *= 0**. The BH procedure of controlling FDR at 5% was applied to the P values of the F test. A cutoff point *α*_*new*_, which is equal to the largest P value considered to be significant, was determined by the above BH procedure. By this procedure, each test for the gene that rejected the F test is at least *α*_*new *_level test.

For each selected gene, a specific sequence of *m*-1 contrasts was tested to determine its expression pattern. To be consistent with the BH procedure performed, the family-wise error rates (FWER) for these genes have to be controlled at least at the level of *α*_*new*_. In this study, we controlled the maximum family-wise error rates (MFWER) by using the Studentized Maximum Modulus distribution [34]. The following theorem in the Appendix outlined the concern of gene-based controlling MFWER at the level of *α*_*new*_.

#### Outline of the analysis

A short summary of the statistical methods used in this study follows:

1. Linear models were used to describe the data based on the experimental design. For each gene, an ANOVA F test was performed based on the described model, and the corresponding P-value was obtained.

2. To adjust for multiple tests based on the large number of genes, the BH method of controlling FDR [22] at 5% was applied to the P-values obtained above, providing a list of genes (list I) that exhibit significant differences among the means of the 5 sampling points.

3. Using *α*_*new*_, which equals the largest P-value determined to be significant in step 2 as the cut-off point, we grouped genes in list I into 4 classes based on the timing of their initial responses by testing the reverse Helmert contrasts sequentially. The Studentized Maximum Modulus distribution parameter *m*-2 = 3 and *v *= 10 were used in this example study, where *α*_*new *_= 0.009545 and |Mαnew,m−2,v|
 MathType@MTEF@5@5@+=feaafiart1ev1aaatCvAUfKttLearuWrP9MDH5MBPbIqV92AaeXatLxBI9gBaebbnrfifHhDYfgasaacH8akY=wiFfYdH8Gipec8Eeeu0xXdbba9frFj0=OqFfea0dXdd9vqai=hGuQ8kuc9pgc9s8qqaq=dirpe0xb9q8qiLsFr0=vr0=vr0dc8meaabaqaciaacaGaaeqabaqabeGadaaakeaadaabdaqaaiabd2eannaaBaaaleaaiiGacqWFXoqydaWgaaadbaGaemOBa4MaemyzauMaem4DaChabeaaliabcYcaSiabd2gaTjabgkHiTiabikdaYiabcYcaSiabdAha2bqabaaakiaawEa7caGLiWoaaaa@3DAB@ = |*M*_0.009545,3,10_| = 3.8651.

4. Using the same critical value |Mαnew,m−2,v|
 MathType@MTEF@5@5@+=feaafiart1ev1aaatCvAUfKttLearuWrP9MDH5MBPbIqV92AaeXatLxBI9gBaebbnrfifHhDYfgasaacH8akY=wiFfYdH8Gipec8Eeeu0xXdbba9frFj0=OqFfea0dXdd9vqai=hGuQ8kuc9pgc9s8qqaq=dirpe0xb9q8qiLsFr0=vr0=vr0dc8meaabaqaciaacaGaaeqabaqabeGadaaakeaadaabdaqaaiabd2eannaaBaaaleaaiiGacqWFXoqydaWgaaadbaGaemOBa4MaemyzauMaem4DaChabeaaliabcYcaSiabd2gaTjabgkHiTiabikdaYiabcYcaSiabdAha2bqabaaakiaawEa7caGLiWoaaaa@3DAB@, we further clustered genes in each of the above classes by testing appropriate contrasts for the subsequent sampling time points.

5. Based on the results of the *m*-1 contrasts for each gene, we also can select genes which share similar pattern of expression for any number of sampling intervals from the beginning.

#### Statistical software

We used the SAS (version 9.0) proc GLM procedure to do model fitting and significance analysis. The SAS program implementing linear models for the olfactory sensory epithelia data is available [38].

### Authors' contributions

HL carried out the statistical analyses and formulation of statistical methods. YL automized the statistical analysis using Splus/R. TVG and MLG carried out the molecular genetics studies. CLW and AJS supervised the study. All authors contributed to the writing of this manuscript. All authors read and approved the final manuscript.

### Appendix

*Theorem *For any balanced one-way model with *m *treatment groups and assuming normality and equal variance *σ*^2^, Let *λ*_1_, *λ*_2_, ..., *λ*_*k *_be an arbitrary complete set of contrasts such that

λi=∑i=1mciμi,i=1,2,…,K
 MathType@MTEF@5@5@+=feaafiart1ev1aaatCvAUfKttLearuWrP9MDH5MBPbIqV92AaeXatLxBI9gBaebbnrfifHhDYfgasaacH8akY=wiFfYdH8Gipec8Eeeu0xXdbba9frFj0=OqFfea0dXdd9vqai=hGuQ8kuc9pgc9s8qqaq=dirpe0xb9q8qiLsFr0=vr0=vr0dc8meaabaqaciaacaGaaeqabaqabeGadaaakeaaiiGacqWF7oaBdaWgaaWcbaGaemyAaKgabeaakiabg2da9maaqahabaGaem4yam2aaSbaaSqaaiabdMgaPbqabaGccqWF8oqBdaWgaaWcbaGaemyAaKgabeaakiabcYcaSiabdMgaPjabg2da9iabigdaXiabcYcaSiabikdaYiabcYcaSiablAciljabcYcaSiabdUealbWcbaGaemyAaKMaeyypa0JaeGymaedabaGaemyBa0ganiabggHiLdaaaa@481A@

Under the null hypothesis, let ***P ***be the distribution of the vector ***λ ***= [*λ*_1_, *λ*_2_, ..., *λ*_*K*_] with mean **0 **and covariance matrix **∑**, let ***P***_***K ***_be the distribution of the vector of a complete set of contrasts λ*=[λ1*,λ2*,…,λK*]
 MathType@MTEF@5@5@+=feaafiart1ev1aaatCvAUfKttLearuWrP9MDH5MBPbIqV92AaeXatLxBI9gBaebbnrfifHhDYfgasaacH8akY=wiFfYdH8Gipec8Eeeu0xXdbba9frFj0=OqFfea0dXdd9vqai=hGuQ8kuc9pgc9s8qqaq=dirpe0xb9q8qiLsFr0=vr0=vr0dc8meaabaqaciaacaGaaeqabaqabeGadaaakeaaiiWacqWF7oaBieqacqGFQaGkcqGH9aqpcqGGBbWwiiGacqqF7oaBdaqhaaWcbaGaeGymaedabaGaeiOkaOcaaOGaeiilaWIae03UdW2aa0baaSqaaiabikdaYaqaaiabcQcaQaaakiabcYcaSiablAciljabcYcaSiab9T7aSnaaDaaaleaacqWGlbWsaeaacqGGQaGkaaGccqGGDbqxaaa@41DF@ with the covariance matrix **∑_*K *_= *Iσ*^2 ^**is the diagonal of **∑ **then the gene based maximum family-wised error rate (MFWER) at any level of *α *of testing a specific sequence of contrasts (list in the Methods) after rejecting the overall F test is achieved by comparing |*T*_*i*_| with |*M*_*α*,*k*-1,*v*_|, where *v *is the df of error,

Ti=λ^iMSE∑i=1mci2n
 MathType@MTEF@5@5@+=feaafiart1ev1aaatCvAUfKttLearuWrP9MDH5MBPbIqV92AaeXatLxBI9gBaebbnrfifHhDYfgasaacH8akY=wiFfYdH8Gipec8Eeeu0xXdbba9frFj0=OqFfea0dXdd9vqai=hGuQ8kuc9pgc9s8qqaq=dirpe0xb9q8qiLsFr0=vr0=vr0dc8meaabaqaciaacaGaaeqabaqabeGadaaakeaacqWGubavdaWgaaWcbaGaemyAaKgabeaakiabg2da9maalaaabaacciGaf83UdWMbaKaadaWgaaWcbaGaemyAaKgabeaaaOqaamaakaaabaGaemyta0Kaem4uamLaemyrau0aaSaaaeaadaaeWbqaaiabdogaJnaaDaaaleaacqWGPbqAaeaacqaIYaGmaaaabaGaemyAaKMaeyypa0JaeGymaedabaGaemyBa0ganiabggHiLdaakeaacqWGUbGBaaaaleqaaaaaaaa@4393@

and |*M*_*α*,*k*-1,*v*_| is the 100(1 - *α*) percentile from the Studentized Maximum Modulus distribution.

*Proof *Let *λ*_1_, *λ*_2_, ..., *λ*_*K *_be any complete set of contrasts, then let

V_0 _= {0 ≤ *i *≤ *K*: *λ*_*i *_= 0} and

V_1 _= {0 ≤ *j *≤ *K*: *λ*_*j *_≠ 0},

let test function

φ(λi)={1if reject the H0i:λi=00if fail to reject the H0i:λi=0
 MathType@MTEF@5@5@+=feaafiart1ev1aaatCvAUfKttLearuWrP9MDH5MBPbIqV92AaeXatLxBI9gBaebbnrfifHhDYfgasaacH8akY=wiFfYdH8Gipec8Eeeu0xXdbba9frFj0=OqFfea0dXdd9vqai=hGuQ8kuc9pgc9s8qqaq=dirpe0xb9q8qiLsFr0=vr0=vr0dc8meaabaqaciaacaGaaeqabaqabeGadaaakeaaiiGacqWFgpGzdaqadaqaaiab=T7aSnaaBaaaleaacqWGPbqAaeqaaaGccaGLOaGaayzkaaGaeyypa0ZaaiqaaeaafaqabeGacaaabaGaeGymaedabaGaeeyAaKMaeeOzayMaeeiiaaIaeeOCaiNaeeyzauMaeeOAaOMaeeyzauMaee4yamMaeeiDaqNaeeiiaaIaeeiDaqNaeeiAaGMaeeyzauMaeeiiaaIaemisaG0aaSbaaSqaaiabicdaWiabdMgaPbqabaGccqGG6aGocqWF7oaBdaWgaaWcbaGaemyAaKgabeaakiabg2da9iabicdaWaqaaiabicdaWaqaaiabbMgaPjabbAgaMjabbccaGiabbAgaMjabbggaHjabbMgaPjabbYgaSjabbccaGiabbsha0jabb+gaVjabbccaGiabbkhaYjabbwgaLjabbQgaQjabbwgaLjabbogaJjabbsha0jabbccaGiabbsha0jabbIgaOjabbwgaLjabbccaGiabdIeainaaBaaaleaacqaIWaamcqWGPbqAaeqaaOGaeiOoaOJae83UdW2aaSbaaSqaaiabdMgaPbqabaGccqGH9aqpcqaIWaamaaaacaGL7baaaaa@7701@

(1) Suppose V_1 _is empty such that *λ*_*i *_= 0 ∀*i*, then MFWER is

MFWER=Pr⁡{at least one false reject}=Pr⁡{(F test rejected)∩(∪i∈V0φ(λi)=1)}≤Pr⁡{F test rejected}≤α
 MathType@MTEF@5@5@+=feaafiart1ev1aaatCvAUfKttLearuWrP9MDH5MBPbIqV92AaeXatLxBI9gBaebbnrfifHhDYfgasaacH8akY=wiFfYdH8Gipec8Eeeu0xXdbba9frFj0=OqFfea0dXdd9vqai=hGuQ8kuc9pgc9s8qqaq=dirpe0xb9q8qiLsFr0=vr0=vr0dc8meaabaqaciaacaGaaeqabaqabeGadaaakeaafaqadeqbbaaaaeaacqWGnbqtcqWGgbGrcqWGxbWvcqWGfbqrcqWGsbGuaeaacqGH9aqpcyGGqbaucqGGYbGCdaGadaqaaiabbggaHjabbsha0jabbccaGiabbYgaSjabbwgaLjabbggaHjabbohaZjabbsha0jabbccaGiabb+gaVjabb6gaUjabbwgaLjabbccaGiabbAgaMjabbggaHjabbYgaSjabbohaZjabbwgaLjabbccaGiabbkhaYjabbwgaLjabbQgaQjabbwgaLjabbogaJjabbsha0bGaay5Eaiaaw2haaaqaaiabg2da9iGbccfaqjabckhaYnaacmaabaGaeiikaGIaeeOrayKaeeiiaaIaeeiDaqNaeeyzauMaee4CamNaeeiDaqNaeeiiaaIaeeOCaiNaeeyzauMaeeOAaOMaeeyzauMaee4yamMaeeiDaqNaeeyzauMaeeizaqMaeeykaKYaaqbaaeaacqGGOaakdaWeqbqaaGGaciab=z8aMjabcIcaOiab=T7aSnaaBaaaleaacqWGPbqAaeqaaOGaeiykaKIaeyypa0JaeGymaeJaeiykaKcaleaacqWGPbqAcqGHiiIZcqWGwbGvdaWgaaadbaGaeGimaadabeaaaSqab0GaeSOkIufaaSqabeqaniablMIijbaakiaawUhacaGL9baaaeaacqGHKjYOcyGGqbaucqGGYbGCdaGadaqaaiabbAeagjabbccaGiabbsha0jabbwgaLjabbohaZjabbsha0jabbccaGiabbkhaYjabbwgaLjabbQgaQjabbwgaLjabbogaJjabbsha0jabbwgaLjabbsgaKbGaay5Eaiaaw2haaaqaaiabgsMiJkab=f7aHbaaaaa@A1D2@

(2) V_0 _is empty such that *λ*_*i *_≠ 0 ∀*i*, then MFWER is 0.

(3) Suppose that neither V_0 _nor V_1 _is empty, then MFWER is

MFWER=Pr⁡{at least one false rejection}=Pr⁡{(F test rejected)∩(∪i∈V0φ(λi)=1)}≤Pr⁡{∪i∈V0φ(λi)=1}≤Pr⁡{∪i∈V0,v0=K−1φ(λi)=1} where v0 is the number of elements in V0 and max⁡(v0)=K−1=1−Pr⁡{∩i∈V0,v0=K−1φ(λi)=0}=1−P{∩i∈V0,v0=K−1[|Ti|≤q]}≤1−PK{∩i∈V0,v0=K−1[|Ti|≤q]} by Sidak's inequality (1967,(8))≤1−PK{max⁡i∈V0,v0=K−1|Ti|≤q}≤1−PK{max⁡i∈V0,v0=K−1|Ti|≤q}=PK{max⁡i∈V0,v0=K−1|Ti|≥q}
 MathType@MTEF@5@5@+=feaafiart1ev1aaatCvAUfKttLearuWrP9MDH5MBPbIqV92AaeXatLxBI9gBaebbnrfifHhDYfgasaacH8akY=wiFfYdH8Gipec8Eeeu0xXdbba9frFj0=OqFfea0dXdd9vqai=hGuQ8kuc9pgc9s8qqaq=dirpe0xb9q8qiLsFr0=vr0=vr0dc8meaabaqaciaacaGaaeqabaqabeGadaaakeaafaqabeqabaaabaqbaeWabSqaaaaaaeaacqWGnbqtcqWGgbGrcqWGxbWvcqWGfbqrcqWGsbGuaeaacqGH9aqpcyGGqbaucqGGYbGCdaGadaqaaiabbggaHjabbsha0jabbccaGiabbYgaSjabbwgaLjabbggaHjabbohaZjabbsha0jabbccaGiabb+gaVjabb6gaUjabbwgaLjabbccaGiabbAgaMjabbggaHjabbYgaSjabbohaZjabbwgaLjabbccaGiabbkhaYjabbwgaLjabbQgaQjabbwgaLjabbogaJjabbsha0jabbMgaPjabb+gaVjabb6gaUbGaay5Eaiaaw2haaaqaaiabg2da9iGbccfaqjabckhaYnaacmaabaGaeiikaGIaeeOrayKaeeiiaaIaeeiDaqNaeeyzauMaee4CamNaeeiDaqNaeeiiaaIaeeOCaiNaeeyzauMaeeOAaOMaeeyzauMaee4yamMaeeiDaqNaeeyzauMaeeizaqMaeeykaKYaaqbaaeaacqGGOaakdaWeqbqaaGGaciab=z8aMjabcIcaOiab=T7aSnaaBaaaleaacqWGPbqAaeqaaOGaeiykaKIaeyypa0JaeGymaeJaeiykaKcaleaacqWGPbqAcqGHiiIZcqWGwbGvdaWgaaadbaGaeGimaadabeaaaSqab0GaeSOkIufaaSqabeqaniablMIijbaakiaawUhacaGL9baaaeaacqGHKjYOcyGGqbaucqGGYbGCdaGadaqaamaatafabaGae8NXdyMaeiikaGIae83UdW2aaSbaaSqaaiabdMgaPbqabaGccqGGPaqkcqGH9aqpcqaIXaqmaSqaaiabdMgaPjabgIGiolabdAfawnaaBaaameaacqaIWaamaeqaaaWcbeqdcqWIQisvaaGccaGL7bGaayzFaaaabaGaeyizImQagiiuaaLaeiOCai3aaiWaaeaadaWeqbqaaiab=z8aMjabcIcaOiab=T7aSnaaBaaaleaacqWGPbqAaeqaaOGaeiykaKIaeyypa0JaeGymaedaleaacqWGPbqAcqGHiiIZcqWGwbGvdaWgaaadbaGaeGimaadabeaaliabcYcaSiabdAha2naaBaaameaacqaIWaamaeqaaSGaeyypa0Jaem4saSKaeyOeI0IaeGymaedabeqdcqWIQisvaaGccaGL7bGaayzFaaGaeeiiaaIaee4DaCNaeeiAaGMaeeyzauMaeeOCaiNaeeyzauMaeeiiaaIaemODay3aaSbaaSqaaiabbcdaWaqabaGccqqGGaaicqqGPbqAcqqGZbWCcqqGGaaicqqG0baDcqqGObaAcqqGLbqzcqqGGaaicqqGUbGBcqqG1bqDcqqGTbqBcqqGIbGycqqGLbqzcqqGYbGCcqqGGaaicqqGVbWBcqqGMbGzcqqGGaaicqqGLbqzcqqGSbaBcqqGLbqzcqqGTbqBcqqGLbqzcqqGUbGBcqqG0baDcqqGZbWCcqqGGaaicqqGPbqAcqqGUbGBcqqGGaaicqWGwbGvdaWgaaWcbaGaeGimaadabeaakiabbccaGiabbggaHjabb6gaUjabbsgaKjabbccaGiGbc2gaTjabcggaHjabcIha4naabmaabaGaemODay3aaSbaaSqaaiabicdaWaqabaaakiaawIcacaGLPaaacqGH9aqpcqWGlbWscqGHsislcqaIXaqmaeaacqGH9aqpcqaIXaqmcqGHsislcyGGqbaucqGGYbGCdaGadaqaamaauafabaGae8NXdyMaeiikaGIae83UdW2aaSbaaSqaaiabdMgaPbqabaGccqGGPaqkcqGH9aqpcqaIWaamaSqaaiabdMgaPjabgIGiolabdAfawnaaBaaameaacqaIWaamaeqaaSGaeiilaWIaemODay3aaSbaaWqaaiabicdaWaqabaWccqGH9aqpcqWGlbWscqGHsislcqaIXaqmaeqaniablMIijbaakiaawUhacaGL9baaaeaacqGH9aqpcqaIXaqmcqGHsislieGacqGFqbaudaGadaqaamaauafabaWaamWaaeaadaabdaqaaiabdsfaunaaBaaaleaacqWGPbqAaeqaaaGccaGLhWUaayjcSdGaeyizImQaemyCaehacaGLBbGaayzxaaaaleaacqWGPbqAcqGHiiIZcqWGwbGvdaWgaaadbaGaeGimaadabeaaliabcYcaSiabdAha2naaBaaameaacqaIWaamaeqaaSGaeyypa0Jaem4saSKaeyOeI0IaeGymaedabeqdcqWIPissaaGccaGL7bGaayzFaaaabaGaeyizImQaeGymaeJaeyOeI0Iaemiuaa1aaSbaaSqaaiabdUealbqabaGcdaGadaqaamaauafabaWaamWaaeaadaabdaqaaiabdsfaunaaBaaaleaacqWGPbqAaeqaaaGccaGLhWUaayjcSdGaeyizImQaemyCaehacaGLBbGaayzxaaaaleaacqWGPbqAcqGHiiIZcqWGwbGvdaWgaaadbaGaeGimaadabeaaliabcYcaSiabdAha2naaBaaameaacqaIWaamaeqaaSGaeyypa0Jaem4saSKaeyOeI0IaeGymaedabeqdcqWIPissaaGccaGL7bGaayzFaaGaeeiiaaIaeeOyaiMaeeyEaKNaeeiiaaIaee4uamLaeeyAaKMaeeizaqMaeeyyaeMaee4AaSMaee4jaCIaee4CamNaeeiiaaIaeeyAaKMaeeOBa4MaeeyzauMaeeyCaeNaeeyDauNaeeyyaeMaeeiBaWMaeeyAaKMaeeiDaqNaeeyEaKNaeeiiaaIaeeikaGIaeeymaeJaeeyoaKJaeeOnayJaee4naCJaeeilaWIaeeikaGIaeeioaGJaeeykaKIaeeykaKcabaGaeyizImQaeGymaeJaeyOeI0Iaemiuaa1aaSbaaSqaaiabdUealbqabaGcdaGadaqaamaaxababaGagiyBa0MaeiyyaeMaeiiEaGhaleaacqWGPbqAcqGHiiIZcqWGwbGvdaWgaaadbaGaeGimaadabeaaliabcYcaSiabdAha2naaBaaameaacqaIWaamaeqaaSGaeyypa0Jaem4saSKaeyOeI0IaeGymaedabeaakmaaemaabaGaemivaq1aaSbaaSqaaiabdMgaPbqabaaakiaawEa7caGLiWoacqGHKjYOcqWGXbqCaiaawUhacaGL9baaaeaacqGHKjYOcqaIXaqmcqGHsislcqWGqbaudaWgaaWcbaGaem4saSeabeaakmaacmaabaWaaCbeaeaacyGGTbqBcqGGHbqycqGG4baEaSqaaiabdMgaPjabgIGiolabdAfawnaaBaaameaacqaIWaamaeqaaSGaeiilaWIaemODay3aaSbaaWqaaiabicdaWaqabaWccqGH9aqpcqWGlbWscqGHsislcqaIXaqmaeqaaOWaaqWaaeaacqWGubavdaWgaaWcbaGaemyAaKgabeaaaOGaay5bSlaawIa7aiabgsMiJkabdghaXbGaay5Eaiaaw2haaaqaaiabg2da9iabdcfaqnaaBaaaleaacqWGlbWsaeqaaOWaaiWaaeaadaWfqaqaaiGbc2gaTjabcggaHjabcIha4bWcbaGaemyAaKMaeyicI4SaemOvay1aaSbaaWqaaiabicdaWaqabaWccqGGSaalcqWG2bGDdaWgaaadbaGaeGimaadabeaaliabg2da9iabdUealjabgkHiTiabigdaXaqabaGcdaabdaqaaiabdsfaunaaBaaaleaacqWGPbqAaeqaaaGccaGLhWUaayjcSdGaeyyzImRaemyCaehacaGL7bGaayzFaaaaaaaaaaa@EB00@

under ***P***_***K***_, based on Sidak's inequality (8) [39], max⁡i∈V0,v0=K−1|Ti|
 MathType@MTEF@5@5@+=feaafiart1ev1aaatCvAUfKttLearuWrP9MDH5MBPbIqV92AaeXatLxBI9gBaebbnrfifHhDYfgasaacH8akY=wiFfYdH8Gipec8Eeeu0xXdbba9frFj0=OqFfea0dXdd9vqai=hGuQ8kuc9pgc9s8qqaq=dirpe0xb9q8qiLsFr0=vr0=vr0dc8meaabaqaciaacaGaaeqabaqabeGadaaakeaadaWfqaqaaiGbc2gaTjabcggaHjabcIha4bWcbaGaemyAaKMaeyicI4SaemOvay1aaSbaaWqaaiabicdaWaqabaWccqGGSaalcqWG2bGDdaWgaaadbaGaeGimaadabeaaliabg2da9iabdUealjabgkHiTiabigdaXaqabaGcdaabdaqaaiabdsfaunaaBaaaleaacqWGPbqAaeqaaaGccaGLhWUaayjcSdaaaa@43B0@ has a Studentized Maximum Modulus distribution [34] with parameter *K*-1 and *v*, let

*MFWER *= α*(*α*, *m *- 1, *v*) = max{*α*(*α*, *m *- 1, *v*)} = *α*,

then *q *= |*M*_*α*,*K*-1,*v*_| is the 100(1-*α*) percentile from above distribution.

## Figures and Tables

**Table 1:** Example of genes from different classesThree genes from each of the 4 classes were selected to illustrate their expression patterns. P_F: P values for the overall F test; P_t: P values at their initial responses; FC: fold changes at their initial responses, where a negative sign indicates down-regulation.

**Class**	**Gene**	**P_F**	**P_t**	**FC**	**Gene Function**
**I**	*Pdcd5*	5.40E-03	6.30E-04	1.2	apoptosis
	*Cetn3*	7.40E-05	3.10E-04	1.2	Ca binding
	*Kit*	3.40E-03	5.30E-04	1.4	growth factor

**II**	*Ccl2*	3.20E-05	3.70E-04	4.1	chemotaxis
	*Csf3*	6.30E-03	5.80E-04	3.2	growth factor
	*Bub3*	2.10E-04	1.20E-04	1.2	cell cycle

**III**	*Omp*	1.40E-04	1.60E-05	-1.6	marker protein
	*Ptdss2*	9.00E-05	8.00E-04	-1.4	enzymatic activity
	*Tfrc*	2.10E-03	3.60E-04	2.1	endocytosis

**IV**	*Casp6*	4.50E-03	6.60E-04	-1.4	Apoptosis
	*Cd68*	4.40E-04	2.90E-05	2	macrophage marker
	*Slfn4*	1.50E-04	7.30E-06	4	cell cycle
